# In Silico and Experimental ADAM17 Kinetic Modeling as Basis for Future Screening System for Modulators

**DOI:** 10.3390/ijms23031368

**Published:** 2022-01-25

**Authors:** Marian Bienstein, Dmitriy Minond, Ulrich Schwaneberg, Mehdi D. Davari, Daniela Yildiz

**Affiliations:** 1 Institute of Biotechnology, RWTH Aachen University, Worringerweg 3, 52074 Aachen, Germany; m.bienstein@biotec.rwth-aachen.de (M.B.); u.schwaneberg@biotec.rwth-aachen.de (U.S.); 2 College of Pharmacy, Nova Southeastern University, Fort Lauderdale, FL 33314, USA; dminond@nova.edu; 3 Rumbaugh-Goodwin Institute for Cancer Research, Nova Southeastern University, Fort Lauderdale, FL 33314, USA; 4 DWI-Leibniz Institute for Interactive Materials, Forckenbeckstraße 50, 52056 Aachen, Germany; 5 Department of Bioorganic Chemistry, Leibniz Institute of Plant Biochemistry, Weinberg 3, 06120 Halle, Germany; 6 Experimental and Clinical Pharmacology and Toxicology, Center for Molecular Signaling (PZMS), Center for Human and Molecular Biology (ZHMB), University of Saarland, Kirrbergerstr., 66421 Homburg, Germany

**Keywords:** ADAM17, metalloproteinases, molecular docking, kinetic modelling, exosite inhibitors, inhibitor design, biocatalysis, reaction mechanism

## Abstract

Understanding the mechanisms of modulators’ action on enzymes is crucial for optimizing and designing pharmaceutical substances. The acute inflammatory response, in particular, is regulated mainly by a disintegrin and metalloproteinase (ADAM) 17. ADAM17 processes several disease mediators such as TNFα and APP, releasing their soluble ectodomains (shedding). A malfunction of this process leads to a disturbed inflammatory response. Chemical protease inhibitors such as TAPI-1 were used in the past to inhibit ADAM17 proteolytic activity. However, due to ADAM17′s broad expression and activity profile, the development of active-site-directed ADAM17 inhibitor was discontinued. New ‘exosite’ (secondary substrate binding site) inhibitors with substrate selectivity raised the hope of a substrate-selective modulation as a promising approach for inflammatory disease therapy. This work aimed to develop a high-throughput screen for potential ADAM17 modulators as therapeutic drugs. By combining experimental and in silico methods (structural modeling and docking), we modeled the kinetics of ADAM17 inhibitor. The results explain ADAM17 inhibition mechanisms and give a methodology for studying selective inhibition towards the design of pharmaceutical substances with higher selectivity.

## 1. Introduction

A disintegrin and metalloproteinases (ADAMs) are multidomain proteins consisting of an N-terminal metalloproteinase domain, a disintegrin domain, an epidermal growth factor (EGF)-like domain, a cysteine-rich domain, a transmembrane domain, and an intracellular C-terminal domain. As ADAM10 and ADAM17 lack the EGF-like domain, the membrane-proximal domain is referred to as stalk region [1]. Both proteases have been shown to contribute to multiple pathophysiological processes, including acute and chronic inflammatory diseases, autoimmune diseases, and cancer formation (for review see [2,3,4]). Furthermore, they are essential for development and homeostasis, highlighted by the fact that the knockout mice are not viable [5,6,7].

There exist several approaches to address ADAMs as therapeutic targets, including targeting expression, maturation, and activation, the use of conformation-specific antibodies, inhibition of the active site by small molecule inhibitors, or changes of the conformation by so-called exosite inhibitors [8,9,10]. Hydroxamate-based compounds, such as TAPI-1 and GI254023X, as small molecule inhibitors chelate the zinc atom in the active site [8,11]. However, most clinical trials failed due to detrimental side-effects by a total inhibition of ADAM proteases and the liver toxicity of the compounds themselves (for review, see [12,13,14]). The disadvantage of side-effects could be avoided by a site-specific application, e.g., inhalation for lung treatment, or the use of substrate-specific inhibitors, which holds at least partly true for exosite inhibitors [15,16]. Despite the substrate specificity, it is essential to elucidate the specific mode of action of ADAM modulators for their evaluation as a therapeutic drug. Precise inspections of the inhibition of the catalytic mechanism, e.g., the distinguishment of a non-competitive inhibitor and an uncompetitive inhibitor and the binding to the active or inactive state, through experiments alone, is difficult [17]. Thus, molecular level simulation is required.

One computational method for identification of potential inhibitor or ligand binding sites and a rough estimation of the relative binding strength is the molecular docking, which is commonly used in different fields, especially in the field of drug development [18,19,20,21]. Docking is mainly used for ligand and binding site identification but can also be used for protein-protein docking [22,23,24]. In the docking process energy minimized small molecules or proteins and the energy minimized target protein are placed in the same simulation cell surrounding the target protein to calculate the binding preferences and its most likely position relative to the target protein. For the binding, all molecular interactions are considered and simulated in different possible conformations. Docking helps to understand the molecular interaction between ligands and receptors and can therefore be used for further binding improvement through ligand modification (drugs) or receptor modification (protein engineering). In addition to that it can also be used for molecular characterization of already engineered proteins [25] or for characterization of protein-protein interactions [26]. Nearly all docking programs and the advantages and disadvantages of docking are well summarized and explained more in detail in [27].

In the current study, we aimed to develop a high-throughput screen for potential ADAM17 modulators and their potential as therapeutic drugs. We built a highly reproducible and easy-to-handle experimental kinetic model for ADAM17 modulators using an artificial substrate and a common cell line. We compared a hydroxamate-based small molecule inhibitor to an exosite inhibitor to evaluate different modes of action. The combination within in silico structural modeling allowed for specific prediction of binding sites of these inhibitors, and the clarification of the mode of action. Thus, we established a screening tool, which can be further translated to a different cell/substrate system. Together with an automated process may be used as a high-throughput assay for drug development.

## 2. Results

### 2.1. Experimental Pharmacology

#### 2.1.1. Experimental Kinetic Modeling Setup

**Figure 1:**
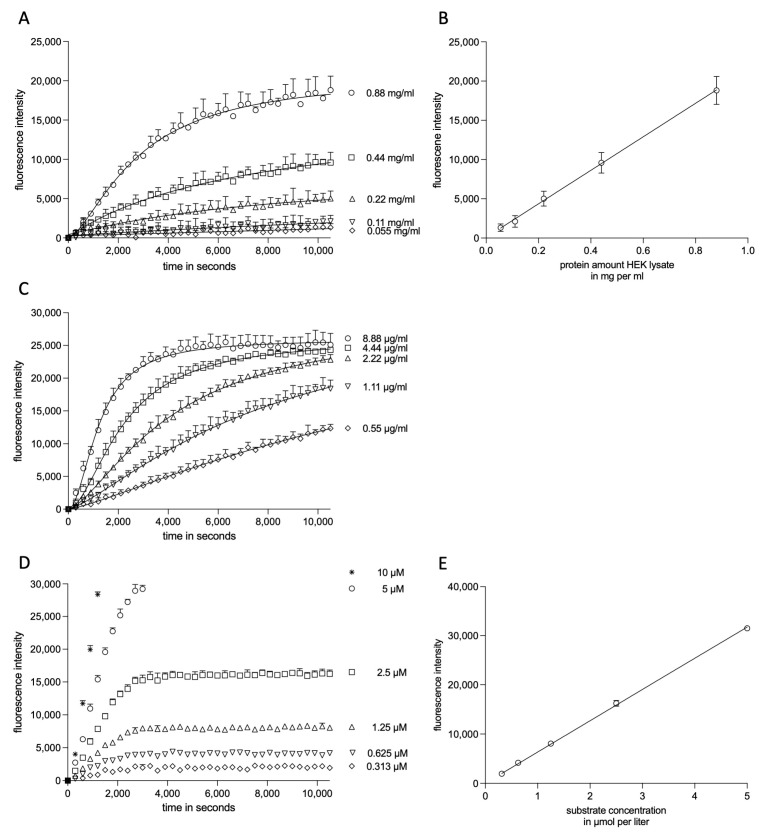
**Experimental kinetic modeling setup.** HEK cell lysates were probed for cleavage of substrate II at varying protein concentrations (**A**, 2.5 µM substrate concentration) and analyzed for linearity (**B**), indicating working at substrate saturation/excess). Determination of total substrate turnover by 1ST3 (**C**, 2.5 µM substrate concentration) and at varying substrate concentrations (**D**) for linear regression (**E**). In (**D**), only data in range are shown (detection limit without baseline subtraction: 50,000). In (**E**), the highest substrate concentration (10 µM) was not implemented as the total substrate turnover could not be achieved/measured. The kinetic measurements (**A**,**C**,**D**) and linear regressions are shown as mean +/± SD of three independent experiments. In (**A**,**C**,**D**), enzyme kinetic velocity fitting was used.

Endpoint measurements have the disadvantage that absolute fluorescence values may vary between different experiments and substrate lots. Kinetic measurements, including the determination of K_M_ and k_cat_, however, would exclude these concerns and ensure the comparability of different experiments. For kinetic measurements with quantification in the linear portion, excess of the substrate is required (referred to as substrate saturation), ensuring that the amount of substrate is not the rate-limiting process. Therefore, we performed kinetic substrate cleavage measurements with varying protein amounts of HEK cell lysates. As the endpoint values after reaching the plateau phase displayed linearity between different protein amounts (Figure 1A,B), it can be assumed that the measurements were performed at substrate excess in the case of HEK cell lysates. Further, a minimum of approximately 0.1 mg/mL total protein was required to detect proteolysis and measure above the detection limit. These facts indicate that the measurements were performed at substrate saturation and that the amount of total protein, including ADAM17 is the rate-limiting process. We next questioned the ratio of substrate turnover and increase in fluorescence. Therefore, we used the well-characterized subtilisin protease 1ST3 [28,29] in our activity assay. Thereby, we could achieve a total substrate turnover as indicated by the same endpoints of the first three protease dilutions (Figure 1C). For linear regression modeling, these experiments were repeated with varying substrate concentrations (Figure 1D), resulting in a linear correlation of fluorescence increase and substrate concentration (Figure 1E).

#### 2.1.2. Kinetic Modeling of ADAM17 Inhibitors

**Figure 2:**
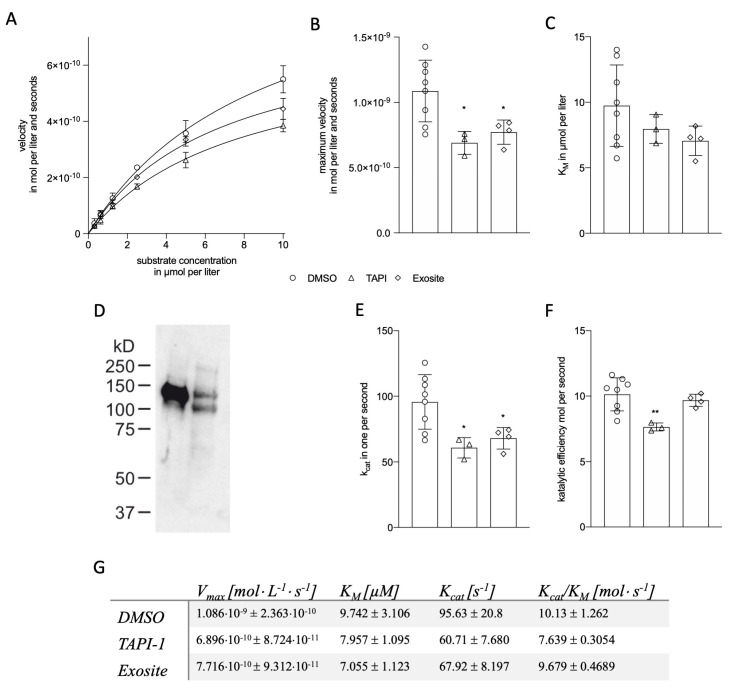
**Kinetic modeling of ADAM17 inhibitors.** To determine V_max_ and K_M_, HEK cell lysates were probed for cleavage of varying substrate concentrations (0 to 10 µM, **A**). V_max_ (**B**) and K_M_ (**C**) were calculated using Michaelis-Menten curve fitting. (**D**) HEK cell lysate (lane 2) was analyzed for the expression of ADAM17 pro-form (130 kDa) and mature form (100 kDa) by Western blot. As a control, HEK cells overexpressing ADAM17 (lane 1) were used. K_cat_ (**E**) and the efficiency (**F**) were calculated from the obtained values. Experimental kinetic data are summarized in (**G**). Data are shown as mean ± SD of at least three independent experiments. Statistical significance was analyzed using one-way ANOVA and Dunnet’s post-hoc test for multiple comparisons (* *p* < 0.05, ** *p* < 0.01).

As mentioned above, determining the mode of action of a potential modulator requires a combination of experimentation and structural modeling. It is well known that TAPI-1 as a hydroxamate-based small molecule inhibitor complexes the Zn ion in the active site of ADAM17. Furthermore, it was described that the exosite inhibitor 2155-17 (complex innovative trial design, CID) (for Ref. see [15]) might bind at a distinct site outside the catalytic center, thereby acting as a modulator of activity. We used these two inhibitors to validate our experimental setup for the prediction of modulator action. We found that both TAPI-1 and the exosite inhibitor significantly reduced the maximum velocity (V_max_), whereas K_M_ remained statistically unchanged (Figure 2A–C,G). In case of the exosite inhibitor, it was experimentally shown to act via non-competitive inhibition [30]. However, we observed a slight reduction of K_M_ (approx. 28%), which did not reach significance and could point toward an uncompetitive or at least mixed-type mode of action.

The inhibitory function of TAPI-1 is exerted by the metal-chelating properties of the hydroxamate residues and thus the binding in the active center. Therefore, one could expect a competitive inhibition of ADAM17 activity by TAPI-1 with competitive inhibition being characterized by an increase of K_M_ with constant V_max_. However, based on our results, it is feasible that both inhibitors may act as non-competitive (no change of K_M_ with decreased V_max_), uncompetitive (reduction of both K_M_ and V_max_) or mixed-type inhibitor, whereas competitive inhibition can already be excluded at this stage due to the kinetic properties [30]. In case of the exosite inhibitor, it was experimentally shown to act via non-competitive inhibition [30]. However, we observed a slight reduction of K_M_ (approx. 28%), which did not reach significance and could point toward an uncompetitive or at least mixed-type mode of action.

#### 2.1.3. Kinetic Modelling as Predictive Tool for ADAM17 Modulators

The effectiveness of an inhibitor can be estimated from the catalytic efficiency (k_cat_/K_M_) of an enzyme. Calculation of the turnover number k_cat_ requires the concentration of the enzyme itself. ADAM17 has two distinct forms: the 130 kDa pro-form carrying the inhibitory pro-domain and the 100 kDa mature form after removing the pro-domain. It was already shown in previous publications that the amount of mature and pro-form ADAM17 equals (for confirmation, see exemplary Western blot in Figure 2D) [31]. Therefore, we measured the concentration of total ADAM17 by ELISA, yielding a final average mature enzyme concentration of 10.22 ± 1.6 pM in HEK cells and the k_cat_ (Figure 2E). Analysis of the catalytic efficiency itself revealed a strong and significant change by TAPI-1, whereas the exosite inhibitor displayed only a minor effect on this value (Figure 2F). K_cat_/K_M_ is a measure for the discrimination of two competing substrates. For TAPI-1, no substrate selectivity has been reported [32]. Therefore, it is feasible that a strong effect on the catalytic efficiency is observed in our kinetic model as the required zinc atom in the active center is chelated. The used exosite inhibitor was shown to inhibit the cleavage of TNFa and heregulin, whereas no effects were reported on Notch, TGFa, betacellulin, CXCL16, and CX_3_CL1 [15]. The catalytic efficiency is strongly dependent on the microenvironment, including charge distribution, and in many cases linked to sequence selectivity and stereoselectivity [33]. Thus, the selectivity reported for the exosite inhibitor seems to be reflected on the catalytic profile, with changes in the velocity and missing changes in the catalytic efficiency. Several FRET-based substrates have been used in activity measurements for ADAM proteases. One example is the Proteolytic Activity Matrix Analysis (PrAMA) [34]. Based on mathematical modeling the relative activity of proteases can be determined. Although these substrates have never been used to determine the catalytic efficiency or the mode of action of ADAM modulators, it seems feasible that these substrates can be easily included and adapted to the current assay. However, with experimental modeling, it is impossible to clearly distinguish between the mode of action (e.g., non-competitive and mixed-type non-competitive and un-competitive) or the binding protease conformation. Thus, for this final step, molecular level simulation is required.

### 2.2. 3D Structure Modeling and Molecular Docking

The prerequisite for molecular docking is the 3D structure of the enzyme, the substrate, and the inhibitor. So far, neither the complete 3D structure of mature ADAM17, including the extracellular, the intracellular and the transmembrane domain, nor the full extracellular domain has not been fully resolved. Only the catalytic domain has been resolved so far. Therefore, modelling the 3D structure of the extracellular and membrane-linked part of ADAM17 was combined to get a structure for exosite inhibitor docking. Furthermore, a docking of this new exosite inhibitor has not been done previously, and the mechanism of a potential exosite inhibitor was not researched yet. The structure of the catalytic domain mainly depends on the resolved 3D structures from different catalytic domains with several substrates.

#### 2.2.1. ADAM17 Structure Modeling

**Figure 3:**
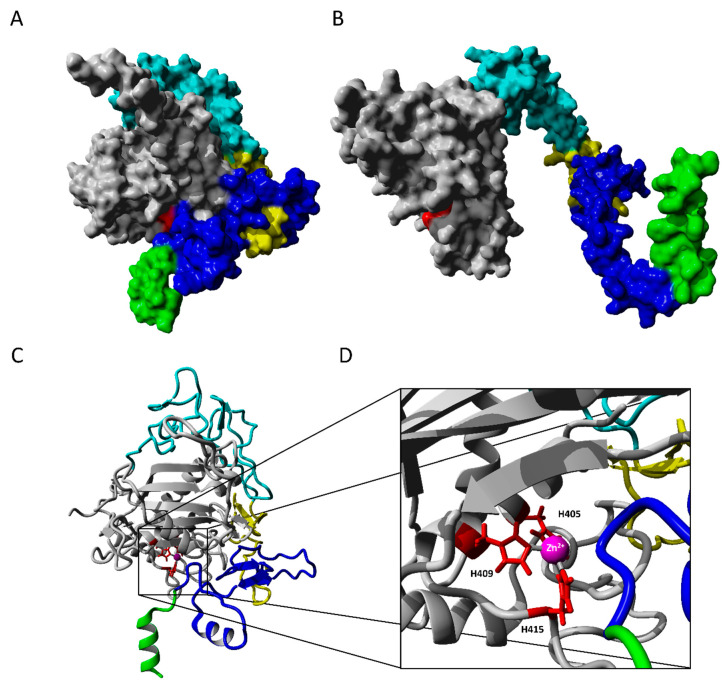
**Structural model of extracellular domain of ADAM17 (R215-W684).** Molecular surface representation and colored subdomains of hybrid model predicted by YASARA (**A**), and extracellular domain model based on AlphaFold Protein Structure Database (**B**); For the extracellular domain of ADAM17 from AlphaFold the full-length ADAM17 model (P78536) was extracted from AlphaFold Protein Structure Database and the intracellular region, the propeptide, and the signal peptide were deleted in YASARA (**C**). Ribbon model with folded linker arm, divided into subdomains (transmembrane: green, membrane-proximal domain: blue, cys-rich: yellow, disintegrin: cyan, metalloprotease: grey); (**D**) A close-up stereo view of the active site taken from (**C**): Zinc ion, bound by His 405, 409 and 415 (red), is marked in magenta.

A hybrid 3D structure of the extracellular domain of ADAM17 was constructed based on multiple templates (Table S1) using YASARA. Z-score of the model structure showed a high-quality model (within range from −3.0 to –3.7, Table S2) and indicated reliability of the model for further molecular docking. In this model, active site residues H405, H409, H415 are coordinated to Zn^2+^ center (Figure 3C). Residues H405 and H409 are located on α-helix, whereas residue H415 resides are located on a loop region (Figure 3C). To examine the stability of predicted protein structures, MD simulation was performed. Protein stability was analyzed using root-mean-square deviation (RMSD) and root-mean-square fluctuation (RMSF) (Figures S1 and S2). RMSD defines the similarity of 3D structures of proteins and the extent of their steadiness. RMSD confirmed the stability of the stability of the predicted hybrid model of the extracellular domain.

The constructed model of the extracellular domain of ADAM17 (R215–W684, Figure 3) included the metalloprotease domain (215–474, grey), the linker arm (475–671), and one part of the transmembrane helix (672–684, green). The linker arm consisting of different domains, including a disintegrin-like domain (475–563, cyan), a cysteine-rich domain (564–602, yellow), and the stalk region (membrane-proximal domain, 603–671), was modeled attached to the globular metalloprotease. In the model, this linker arm is mainly attached via hydrophobic interactions to the catalytic domain (Figure 3A). It lacks interaction at the opposite side from the substrate-binding site, building a small cleft. In nature, the arm would probably work as a free moving linker between membrane and metalloprotease domain as shown in the model from AlphaFold Protein Structure Database [35] (Figure 3B). When modeling with AlphaFold 2.0 of the extracellular domain on its own was applied (R215–W684), the model was more globular (not shown), comparably to the hybrid model of the extracellular domain (Figure 3A).

#### 2.2.2. Molecular Docking

As mentioned above, based on experimental data, it is quite difficult to distinguish between a non-competitive (binding to a site distinct from the active site/substrate binding pocket) and weak uncompetitive action (binding only enzyme-substrate complexes) of inhibitors or to distinguish a non-competitive inhibitor from mixed type inhibitors. Therefore, molecular docking simulations with the extracellular domain and the catalytic domain of ADAM17 were performed. As ligands, TAPI-1, the secretase substrate II and the proposed exosite inhibitor CID17 were used. The exosite inhibitor were used for blind docking, where the exact docking site is not known. In this way, a possible exosite could be determined. For docking of hydroxamate-based inhibitors like TAPI-1, the binding site is already known and docking site could be specified around the zin ion (10 Å around zinc ion) [36,37]. Furthermore, the relative orientation and position from cocrystallized inhibitors (TAPI-2) in similar enzyme structures (TACE) was taken as comparison and verification. Method of docking verification is further described in Section 4.2.3.

#### 2.2.3. TAPI-1 and Catalytic Domain

**Figure 4:**
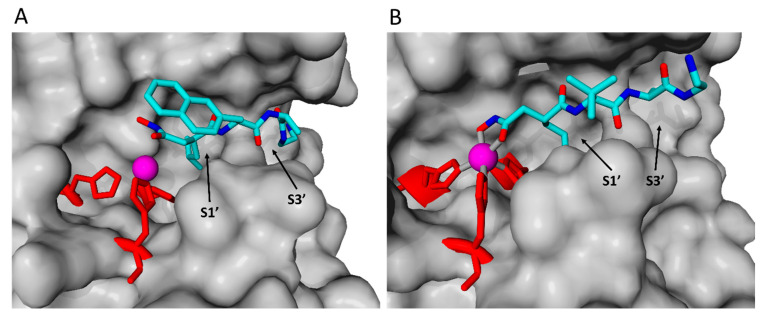
**Comparison of docked TAPI-1 with cocrystallized TAPI-2 (2DDF).** TAPI-1 (**A**) was docked in ADAM17 catalytic domain (Autodock vina & default parameters), while TAPI-2 (**B**) was cocrystallized in TACE (PDB: 2DDF [37]). Hydrogens were deleted after docking for better visual comparison. Zinc (magenta) was bound by three histidines (red) and hydroxymate group was positoned next to the zinc ion. Isobutyl group was binding in S1′ binding pocket, while rest of the inhibitor except of the naphtyl group was bound to S3′.

TAPI-1 is a well-described inhibitor that supposedly binds with its hydroxamate group toward the zinc ion, thereby chelating the metal ion leading to inhibition of the proteolytic activity [38]. The exact binding mechanism of hydroxamate group-based inhibitors is also well described [39]. Furthermore, the specific binding site of hydroxamate-based TACE inhibitors were already identified in [36,37]. Therefore, TAPI-1 was used to validate our modeling and docking strategy with the catalytic domain first. Docking poses were clustered and the first cluster were used for further analysis (Figure 4 and Figure S3). Its calculated binding energy was −7.41 kcal/mol with a dissociation constant of 3733043 pM. The experimental modeling revealed the action of TAPI-1 as a non-competitive, uncompetitive, or mixed type inhibitor. Molecular docking showed that TAPI-1 fits into the substrate-binding site next to the bound zinc ion. Position and relative orientation of TAPI-1 were compared to cocrystallized TAPI-2 in TACE [36,37] (Figure 4). The structural comparison between the inhibitors TAPI-1 and TAPI-2 can be found in the supporting information (Figure S4). The hydroxamate group was directing to the zinc ion, while the isobutyl group was binding in the deep hydrophobic S1′ pocket. The long chain in TAPI-1 on the opposite side of the hydroxamate group was binding to the S3′ binding pocket (Figure 4). The relative orientation and the binding site of TAPI-1 to ADAM17 catalytic domain was very similar to TAPI-2, cocrystallized in TACE (PDB: 2DDF [37]; PDB: 1BKC [36]). For a detailed docking pose of TAPI-1 with all contact residues please refer to Figure S3 in Supporting Information.

Per definition, un-competitive inhibitors (reduction of K_M_ and V_max_) are only bound after initial substrate binding. Consecutive docking revealed that TAPI-1 is not able to enter the active site due to the bulkiness of an already bound substrate. Thus, in line with the lack of statistical significance of the observed slight K_M_ changes, an action as an un-competitive inhibitor can be excluded based on this model. Non-competitive inhibitors bind at sites distinct from the substrate binding’s site. Mixed-type inhibitors differ from non-competitive inhibitors by decreasing the enzyme’s binding affinity for the substrate upon inhibitor binding and vice versa. This is well in line with the fact that a pre-incubation is required in experimental settings, with TAPI-1 potentially binding while the substrate is constitutively associating and dissociating. One could argue that mixed-type inhibitors bind to an allosteric site. As evident from the docking, TAPI-1 binds (a) to one edge of the substrate-binding site, and (b) functions as chelator, thereby disabling for catalysis. Thus, based on the in silico and kinetic modeling, we assume that TAPI-1 is a mixed type non-competitive/competitive inhibitor.

#### 2.2.4. Substrate II, Extracellular and Catalytic Domain of ADAM17

**Figure 5:**
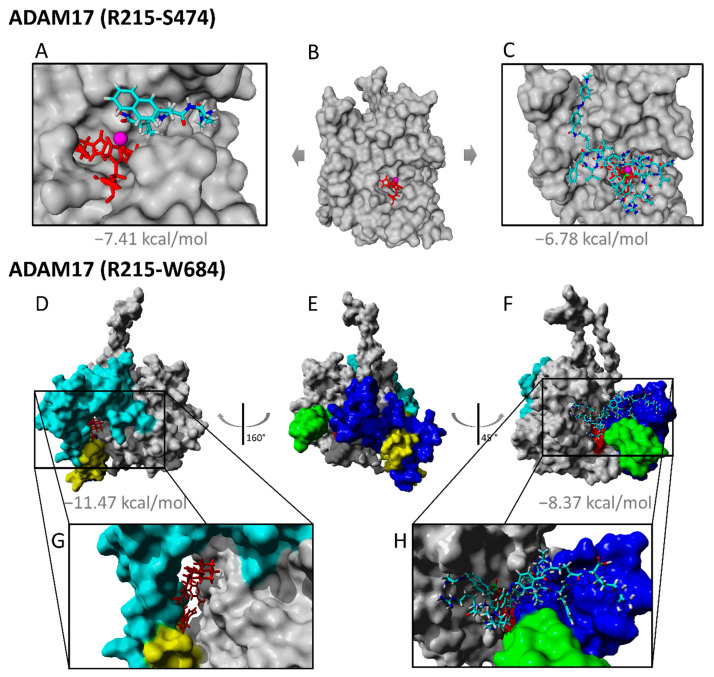
**Molecular docking of substrate and inhibitors with ADAM17 catalytic domain (R215–S474) and ADAM17 extracellular domain (R215–W684).** The catalytic domain (**B**) was docked with TAPI-1 (**A**) and secretase substrate II (**C**), which both bind to the substrate-binding site. TAPI-1 binds with its hydroxamate group to zinc ion (magenta), bound by the Histidines H405, H409, H415 (red). Binding energies are given below each docking pose (grey). Extracellular domain of ADAM17 (**E**) was docked with exosite inhibitor (**D**,**G**) and secretase substrate II (**F**,**H**), where. Black boxes mark zoomed regions (**D**–**F**,**H**). Exosite inhibitor binds on the opposite side of substrate binding site between the disintegrin domain (cyan), the cys-rich (yellow) domain and the catalytic domain (grey surface), while substrate binds partially to the blocked binding site. Zinc is hidden behind the membrane-proximal domain (blue) and helix domain (green).

To compare the binding of an artificial substrate (secretase substrate II; used in the experimental model; chemical structure in supporting information (Figures S5 and S6)) with the extracellular domain and the catalytic domain only, the catalytic domain was modeled as written above. Docking of the artificial substrate was done with the catalytic domain (R215–S474) only (Figure 5C) and in addition with the extracellular domain of ADAM17 (R215–W684) (Figure 5F,H). TAPI-1 served as a comparison (Figure 5A). Substrate docking for the catalytic domain shows a stretched and ordered substrate laying in the binding pocket. Its predicted cleavage site (HQK|LVF) [40] was directly located next to the bound zinc ion in the binding pocket. The substrate was held mainly by hydrophobic interactions. Additionally, one π-π, one cation-π, and three hydrogen bonds could be determined for substrate docking, which resulted in the end in the binding energy of −6.78 kcal/mol. The most favored binding site of the substrate secretase II for docking with the complete extracellular domain was also the substrate-binding site (next to the bound Zinc ion). The predicted cleavage site stayed next to the zinc ion, but binding site was partially blocked by the membrane proximal domain, which also influences the substrate binding by hydrophobic interactions (Figure 5E,F). This leads to final binding energy of −8.37 kcal/mol. A detailed list of all contact residues of substrate docking can be found in SI (Table S9).

When the docking of the substrate with the extracellular domain of ADAM17 (R215–W684) was carried out, the substrate had a more compact form and was not as stretched and ordered (Figure 5F,H) as within the docking with ADAM17 catalytic domain (R215–S474) (Figure 5C) Compared to the other models, the substrate had a higher binding energy of −8.37 kcal/mol and a dissociation constant of 728,634 pM while docking with the extracellular domain of ADAM17 with its linker. A detailed list of contact residues can be found in SI (Table S3).

#### 2.2.5. Exosite Inhibitor and the Extracellular Domain of ADAM17

**Figure 6:**
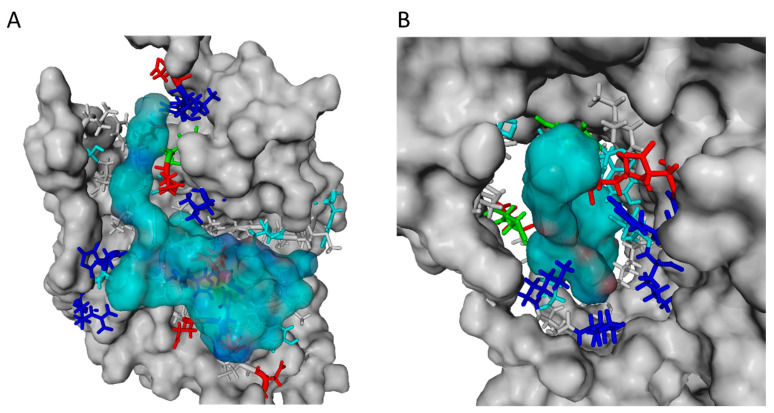
**Molecular docking of secretase II substrate with catalytic domain and CID with extracellular domain of ADAM17.** (**A**) Secretase II substrate (light blue molecular surface) docked to catalytic domain in the substrate binding site (grey molecular surface); (**B**) Exosite inhibitor CID (light blue molecular surface) docked to secondary binding site of extracellular domain of ADAM17 (grey molecular surface). The ligand surface coloring indicates the atoms oxygen (red), nitrogen (blue) and carbon (cyan). All contact residues of the receptor are colored as follows: Positive and negative charged amino acids are shown in blue and red, respectively. Residues in cyan mark AAs with hydroxyl groups, which can form hydrogen bonds. AAs in green mark polar uncharged amino acids. Hydrophobic and special AAs like Cys, Gly, and Pro are shown in grey.

The hypothesis here is that the exosite inhibitor (CID17) binds to the extracellular domain or the outer layer of the metalloproteinase domain of ADAM17, changing its structure or folding to induce an inhibition without chelating the zinc like TAPI-1. Docking the exosite inhibitor with the extracellular domain of ADAM17 model showed a favored docking of inhibitor between the linker arm and the globular form of the catalytic domain (−11.47 kcal/mol) (Figure 5D,G). The exact chemical structure of CID can be found in the supporting information (Figures S7 and S8). A dissociation constant of 3945.0 pM was calculated. In the following, this cleft will be named as exosite. CID17 was mainly bound through many hydrophobic interactions (Figure 5G and Figure 6B). Contact residues were further analyzed and visualized by LigPlot+ [41] (Figure S9). The adamantyl group adds a sterically big hydrophobic and rigid region to the inhibitor. Besides all the positives effects of adamantyl groups [42], this may also help to enter and to stay in the allosteric binding site. Among others hydrophobic groups the used adamantyl group in the exosite could positively influence the future drug design and shorten the drug screening. The binding of exosite inhibitor works possibly like a contact bridge between the linker arm and the catalytic domain (disintegrin domain, catalytic domain), refolds and stabilizes the globular form by attaching the linker more to the catalytic domain (Figure 5D,E,G).

This conformational change would lead to a blocked binding site and a constrained catalytic domain to the cell membrane and, therefore, a less attractive binding site to bulky substrates like TNF. While docking, another binding cluster compared to the named exosite showed the inhibitor bound to one part of the substrate-binding site and the proximal-membrane domain with a binding energy of −10.51 kcal/mol and a dissociation constant of 19,739.4 pM. Here the inhibitor was bound by hydrophobic interactions and three hydrogen bonds. Because of another bridging effect between the stalk region and the catalytic domain, binding of the inhibitor to the substrate site and the stalk region could also stabilize the arm next to the catalytic domain and block the binding pocket simultaneously. Both mechanisms would, in addition, lead to an inaccessible zinc ion/binding site for substrate stabilization/cleavage. This substantiates the hypothesis of an allosteric inhibitor, which does not function via zinc chelating, but through structural changes. As shown by the molecular docking, CID17 preferably binds to an exosite but can also bind to the active center, where it connects proximal-membrane domain and the catalytic domain. Together with the experimental kinetics, where V_max_ was reduced, whereas K_M_ was not changed (Figure 2), we can conclude that CID17 functions as a non-competitive inhibitor, although a binding to the substrate site is possible according to docking.

## 3. Discussion

Regulating human extracellular proteases responsible for shedding is one meaningful way to keep human health and the immune system intact [7,8,11,43,44,45,46]. More specifically, one way of regulating human proteases could be efficient and target-specific inhibitors, like exosite inhibitors. Exosite inhibitors can indirectly modulate proteases through binding at a secondary site and a following conformational change [15,47]. The found exosite inhibitor CID17 (compound 17 in [15]) showed a non-competitive mode of action, where the V_max_ was reduced, and K_M_ remained nearly the same. This mode of inhibition was also described for another metalloprotease inhibitor in 2011 [48]. The high specificity of the inhibitor CID17 compared to other promising inhibitors was summarized in [12]. In addition to that, the computational docking was able to confirm the non-competitive mode of inhibition.

Furthermore, the computational modeling (YASARA, [49]) and the AlphaFold Protein Structure Database could show the difference between a globular form which is automatically preferred in the homology model to decrease the energy, and the more open form, where the linker is not attached to the catalytic domain. This underlines the possibility of two different states, which an exosite inhibitor could regulate. Docking of CID17 showed a favored docking position on the opposite side of the binding site, which is directly connected to the linker arm. One could deduce that the missing hydrophobic interactions that were completed with the inhibitor lead to an attachment of the linker arm through additional hydrophobic interactions, which finally hinders the substrate binding in the substrate-binding region. Besides the allosteric site, CID17 was also bound to the substrate site and connected the linker and the catalytic domains while also blocking the substrate-binding site. We previously described a structurally related ADAM17 inhibitor that can also potentially bind across domains due to the spatial proximity of the non-catalytic and catalytic domains, which corroborates with the evidence presented herein [30]. In another study, a very promising inhibiting antibody “D1” binds at a similar position as the CID17 s most favored cluster, experiments showed [50]. Here, the inhibition was explained by blocking the substrate site itself on a molecular level. However, the binding of the antibody occurred across the domains, which could also help block the site via the linker domain. Comparing the results with the state of the art, this inhibition mechanism could be a new approach for tailored drug development for ADAM17 inhibition as the effect on the binding of single substrates and the subsequent cleavage could be predicted. New developed inhibitors should target not the zinc ion anymore, but more the allosteric site. Here, the potential computational screening of small molecules is the next step to optimize the binding of these shown exosite inhibitor. Observation of CID docking showed that especially the sterically large conformation and the hydrophobic patches play an in important role in this bridging effect. Starting from here, new exosite drugs can be designed by modifying CID or structural similar compounds [15]. This understanding would significantly influence tackling chronic and life-threatening diseases, which were found to be connected to ADAM17, such as cancer and chronic inflammatory diseases like rheumatoid arthritis [12,51,52,53,54,55]. Recently COVID19 was also associated with an ADAM17 malfunction, which further underlines the current importance of understanding the inhibition mechanism of ADAM17 in detail for external regulation through substrate-specific inhibitors [56,57,58,59,60].

## 4. Materials and Methods

### 4.1. Experimental Pharmacology

All compounds were >95% pure by HPLC.

#### 4.1.1. Cell Culture, Sample Preparation and Cleavage Assay

Human embryonic kidney cells were cultured in DMEM high glucose supplemented with 10% FBS and sub-cultured at 80% confluence. Cells were lysed in extraction buffer (0.15 M sodium citrate, 1% Tween 80, pH 8.0) and centrifuged for 10 min at 16,000× *g* and 4 °C. The protein content in the supernatant was determined by Nanodrop (Thermo Scientific, Germany) and adjusted to 0.8 mg/mL. It is important to note, that the final protein concentration should be at least 0.5 mg/mL. The samples were two-fold diluted with reaction buffer (25 mM Tris-HCl, 0.01% Triton X-100, pH 8.0) and pre-incubated with either inhibitors (10 µM) or vehicle control (0.1% DMSO). The fluorogenic alpha-secretase substrate II (565767, Sigma-Aldrich, Darmstadt, Germany) was added to final concentrations as indicated in the figure legends, and subsequently the increase in fluorescence (excitation 355 nM, emission 510 nm) was measured each 5 min for 36 cycles at 37 °C using a Tecan plate reader (Tecan Trading AG, Zürich, Switzerland). In case of the subtilisin protease 1ST3, protein concentration was determined by bicinchoninic acid (BCA) assay (10741395, ThermoFisher, Dreieich, Germany) and used at a final concentration of 8 µg/mL. As a linearity control, a two-fold dilution series of the initial protein extract or 1ST3 was measured in parallel. To exclude effects of varying substrate lot numbers, background activity and bleaching processes on the experimental results, a blank control containing no lysate was measured in parallel and subtracted in each experiment. The maximum velocity (V_max_) and the concentration at half-maximal velocity (Michaelis-Menten constant, K_M_) were determined by enzyme kinetic modeling. The mode of action was determined in a combination of experimentation and structural modelling with the following assumption: non-competitive inhibition, constant K_M_ with reduced V_max_; competitive inhibition, increased K_M_ with constant V_max_; un-competitive inhibition and mixed-type models, reduced K_M_ with reduced V_max_.

#### 4.1.2. Elisa Measurement

The concentration of ADAM17 in cell lysate was determined by ELISA using a commercial kit (DY930, R&D Systems, Wiesbaden, Germany). Different dilutions of cell lysates were measured to check for accuracy and linearity.

#### 4.1.3. Western Blot

HEK cell lysates were probed for ADAM17 pro-form and mature form expression as described before [31]. Briefly, lysates were subjected to SDS-PAGE and proteins were transferred to nitrocellulose membrane (Amersham, UK). Membranes were blocked with 5% (*w*/*v*) non-fat dry milk in tris buffered saline with 0.05% Tween for 1 h and probed overnight at 4 °C against ADAM17 (1 µg/mL, rabbit polyclonal antibodies against C-terminus, Millipore, Darmstadt, Germany), followed by incubation with a POD-coupled secondary antibodies (diluted 1:20.000). After addition of the chemiluminescent substrate (Perkin Elmer, Waltham, MA, USA), signals were recorded using a luminescent image analyzer LAS3000 and quantified using AIDA image analyzer v.4.27 (Elysia-raytest GmbH, Straubenhardt, Germany).

#### 4.1.4. Expression and Purification of 1ST3

The expression of 1ST3 with *Bacillus subtilis (B. subtilis)* DB104.1 was done in three 500 mL flasks with each 100 mL buffered LB-Media (pH 8.6) (1% (*m*/*v*) tryptone, 0.5% (*m*/*v*) yeast extract and 1% (*m*/*v*) sodium chloride) supplemented with kanamycin (100 µg/mL). After culturing for 72 h at 250 rpm and 37 °C, bacterial cultures were centrifuged at 14,000× *g* and the supernatant was transferred for further use. 250 mL of the supernatant were dialyzed against 20 mM HEPES (pH 7) overnight. The dialyzed fraction was then loaded on a 5 mL cationic exchange chromatography column (GE Healthcare HiTrap SP HP) with a flow rate of 2 mL/min. The protease was eluted via gradient (4 mL/min) with 20 mM HEPES (pH 8.0) supplemented with 1 M NaCl. The purified enzyme (47 mL) was again dialyzed against 75 mM sodium citrate buffer with 12.5 mM Tris-HCl, 0.005% Triton X-100 at pH 8.0. The expression resulted in 47 mL purified enzyme and 50 mL unpurified enzyme in buffered LB-media. Protein concentration was determined by bicinchoninic acid (BCA) assay (10741395, ThermoFisher, Dreieich, Germany) and used at a final concentration of 8 µg/mL.

### 4.2. 3D Protein Structure Modeling and Molecular Docking

#### 4.2.1. Structural Analysis

The protein sequence of human a disintegrin and metalloproteinase 17 protein (ADAM17, gene ID: 6868, NP_003174.3), which consists of 824 amino acids (AAs), was retrieved from the National Center for Biotechnology Information (NCBI) at http://www.ncbi.nlm.nih.gov/protein (accessed on 11 January 2022) (sequence is provided in Supplementary information (Text S1)). The first 214 amino acids, which comprise the pro-peptide and the signal peptide are cleaved while maturing. Referring to Uniprot [61] (P78536) the mature enzymes’ topology can be divided into three regions, the extracellular domain (215–671), the transmembrane helix (672–692) and the cytoplasmic domain. Protein BLAST analysis in the 3D protein structure database showed that neither the 3D protein structure of mature ADAM17 (full length), nor the extracellular domain is resolved experimentally. In contrast to the full protein and the extracellular domain, there are several resolved structures describing the catalytic domain with different inhibitors [37,62,63,64,65,66,67,68,69,70,71,72,73,74]. It was observed that residues 215–672 belong to extracellular domain which is the potential domain for therapeutic targeting (Text S2). From sequence of extracellular domain, the sequence for catalytic domain was extracted (R215–S474) (Text S3). Sequence of extracellular domain and first amino acids of the transmembrane helix of ADAM17 (215–684 AAs) were used for 3D protein structure modeling using YASARA Structure version 17.8.19 [75]. The extracellular domain was chosen because of the hypothesis that not only the catalytic domain, but also the other extracellular regions have an influence on the inhibition process. This extracellular domain model, including part of the transmembrane helix, will be called the extracellular domain in the following. On the other side the catalytic domain (R215–S474) was modelled using the YASARA software package (YASARA Structure version 20.10.4) [75]. Quality of model structures were evaluated according to statistical parameters of YASARA protocol [75] to select the best model for molecular docking study. Further analysis was done with PROCHECK [76]. Detailed evaluation of the model is given in the Supporting Information (Table S2). For comparison of our extracellular domain model the newly established AlphaFold 2.0 structure database was used to extract the extracellular domain out of the full-length protein (P78536), which was modeled by AlphaFold 2.0 [35].

#### 4.2.2. MD Simulation

To validate the predicted hybrid extracellular domain model, MD simulation in YASARA was performed as follows. For preparation the model was neutralized and solvated in a periodic box containing TIP3P [77] water and 0.9% (low ionic strength) NaCl. The MD simulation were performed in triplicate using AMBER14 force field [78,79], and YASARA software package (YASARA Structure version 17.8.19) [75,80,81]. The simulation parameters were kept at the default values defined by the macro. Electrostatics were calculated using a cut-off of 7.86 Å; long-range interactions were calculated by using the particle-mesh Ewald integration. Bond length to hydrogen atoms and bond angles in water were constrained to speed up the simulation [77]. After initial minimization by steepest descent and simulated annealing until convergence (<0.02 kJ mol^−1^ per atom during 200 steps) were reached. MD simulation was performed for 50 ns at 298 K by rescaling the time-averaged atom velocities using a Berendsen thermostat [81] and a solvent density of 0.997 g L^−1^. Snapshots were taken every 25 ps and the recorded trajectories were statistically analyzed using YASARA. RMSD and RMSF can be found in Supporting Information (Figures S1 and S2).

#### 4.2.3. Molecular Docking

Molecular docking of substrate and inhibitor was carried out using VINA implemented in the YASARA software package [75,82]. Docking was performed by using YASARA docking macro, which was modified regarding its runs from 25 to 100 runs. All other parameters were used as default. Receptor as well as ligand were prepared for docking with initial energy minimization experiment, implemented in YASARA. Docking of two ligands was performed consecutively with another minimization step in between. Substrate docking with ADAM17 (extracellular domain and catalytic domain) was carried out using artificial substrate secretase substrate II (Ac-RE(EDANS)-VHHQKLVF-K(DABCYL)-R-OH), used for tumor necrosis factor-α converting enzyme (TACE) as described previously [83] (Figures S5 and S6). The exosite inhibitor molecule in comparison to TAPI-1 was selected based on our previous reports [15] (Figures S7 and S8). For docking of TAPI-1 the simulation cell was constructed 10 Å around the bound zinc ion. For all the other dockings the simulation cell was constructed around all atoms with a distance of 5 Å to do a blind docking for identification of a not known docking site. Docking method was validated by comparison to the cocrystallized inhibitor TAPI-2, cocrystallized in TACE (PDB: 2BKC, 2DDF) [36,37]. For comparison of docked TAPI-1 to cocrystallized TAPI-2 the same position and relative orientation was found in the first cluster docking. In addition to that another docking algorithm, Autodock with Lamarckian Genetic Algorithm (LGA) [84], was used in YASARA to include zinc-oriented atom parameters as described in [85] as a second verification. For this purpose, the before mentioned docking macro in YASARA was adjusted to AutodockLGA and AD4Zn.dat (custom zinc parameters) was used as the parameter file for construction of the grid maps [85], resulting in the same found docking site for TAPI-1, with similar orientation poses. As a third verification, the command line based Autodock4.2 method with zinc customized parameters was used [85,86].

### 4.3. Statistical Analysis

Quantitative data are shown as mean +/± SD calculated from at least three independent experiments and cell isolates. The data were analyzed using PRISM 8.2 (GraphPad Software, La Jolla, CA, USA). A *p*-value < 0.05 was considered significant. Details are included within the figure legends.

## 5. Conclusions

In summary, we modeled the complete extracellular domain of ADAM17 and the catalytic domain, which were docked with an artificial substrate and two different types of inhibitors (TAPI-1 and exosite inhibitor). Through the combination of in silico and experimental and kinetic modeling, we differentiated between distinct modes of inhibitor action distinguishing subtypes of mixed type inhibitors. Furthermore, we found a folding mechanism with the exosite inhibitor, which blocks the binding site and leads to inhibition in this way. We uncovered how the catalytic reaction functions, the charge distribution during cleavage, and the Zinc ion participation in this charge distribution. Thus, the proposed model could be used to screen, e.g., sizeable natural compound libraries for selective inhibitors, which would be of interest for several diseases, including viral and bacterial infections. Nevertheless, for the final validation of an inhibitor, the system would have to be tested in a ‘total cell’ setting resembling the membrane-bound form of ADAM17 and the respective substrate.

## Data Availability

The data presented in this study are available in the article and Supplementary Materials.

## Supplementary Materials

The following supporting information can be downloaded at: https://www.mdpi.com/article/10.3390/ijms23031368/s1.

## Abbreviations

**Abbreviation**	**Term**
ADAMs	A disintegrin and metalloproteinases
Ac-REEDANS-VHHQKLVF-KDABCYL-R-OH	Alpha Secretase Substrate II
AA	Amino acid
APP	amyloid precursor protein
BCA	Bicinchoninic acid
CID17	Exosite inhibitor
GI	GI254023X
IPF	Idiopathic pulmonary fibrosis
LGA	Lamarckian Genetic Algorithm
MP	Metalloproteinase
NCBI	National Center for Biotechnology Information
PrAMA	Proteolytic Activity Matrix Analysis
TACE	Tumor necrosis factor-α converting enzyme
TNF	Tumor necrosis factor-α
YASARA	Yet Another Scientific Artificial Reality Application
