# Supplementary material for: In Silico and Experimental ADAM17 Kinetic Modeling as Basis for Future Screening System for Modulators
Source: Int J Mol Sci. 2022 Jan 25;23(3):1368. doi: 10.3390/ijms23031368 (PMC8835787; doi:10.3390/ijms23031368)
Supplement: Supplementary file 1 [file ijms-23-01368-s001.zip › ijms-1536027-supplementary.pdf]

Supplementary Information

In silico and experimental ADAM17 kinetic modeling as basis for future screening system for modulators

Marian Bienstein[1], Dmitriy Minond[2,3], Ulrich Schwaneberg[1, 4], Mehdi D. Davari[5*], Daniela Yildiz[6*]

[1]Institute of Biotechnology, RWTH Aachen University, Worringerweg 3, 52074 Aachen, Germany

[2]College of Pharmacy, Nova Southeastern University, FL 33314, USA

[3]Rumbaugh-Goodwin Institute for Cancer Research, Nova Southeastern University, FL 33314, USA

[4]DWI-Leibniz Institute for Interactive Materials, Forckenbeckstraße 50, 52056 Aachen, Germany

[5]Department of Bioorganic Chemistry, Leibniz Institute of Plant Biochemistry, Weinberg 3, 06120 Halle, Germany

[6]Experimental and Clinical Pharmacology and Toxicology, Center for Molecular Signaling (PZMS), Center for Human and Molecular Biology (ZHMB), University of Saarland, Kirrbergerstr., 66421 Homburg, Germany

*equal contribution, corresponding authors:

mehdi.davari@ipb-halle.de

daniela.yildiz@uks.eu

**Content**	**Page**
**Table S1: Templates used for hybrid homology modelling of extracellular domain**	S3
**Table S2: Evaluation of quality of ADAM17 homology models**	S3
**Figure S1: RMSD of extracellular domain model for 50 ns**	S4
**Figure S2: RMSF of extracellular domain model for 50 ns**	S4
**Figure S3: Molecular docking of TAPI-1 with the catalytic domain of ADAM17**	S5
**Figure S4: Hydroxamate-based inhibitors TAPI-1 and TAPI-2**	S5
**Figure S5: Structure of secretase substrate II**	S6
**Figure S6: 3D structure of secretase substrate II**	S6
**Figure S7: Structure of inhibitor molecule (CID17)**	S7
**Figure S8: 3D structure of inhibitor molecule (CID17)**	S7
**Table S3: Docking contact residues for different receptors and the used ligands**	S8
**Figure S9: Visualization of key interactions towards bound inhibitor in exosite**	S9
**Text S1: Full-length sequence of human ADAM metallopeptidase domain 17 protein**	S10
**Text S2: Extracellular domain sequence of human ADAM metallopeptidase domain 17 protein**	S10
**Text S3: Catalytic domain sequence of human ADAM metallopeptidase domain 17 protein**	S11
**References**	S12

Table S1: Templates used for hybrid homology modelling of extracellular domain. Modelling was performed with YASARA Structure version 17.8.19 [1]. Templates were taken from YASARA modelling report and are based on previous alignments, done in the process of YASARA modelling.

Template	Transfer	First residue	Last residue	Length (AAs)	Z-Score
6BE6-D	1	1	439	439	**-3.661**
6BE6-D	2	427	470	44	**-3.457**
2I47-B	3	105	117	13	**-3.411**
2I47-B	4	185	245	61	**-3.362**
2I47-B	5	71	94	24	**-3.331**
2I47-B	6	16	30	15	**-3.292**
2I47-B	7	68	166	99	**-3.257**
3LEA-A	8	185	195	11	**-3.246**
3LEA-A	11	1	20	20	**-3.231**
3LEA-A	12	185	195	11	**-3.227**
2I47-B04	14	43	60	18	**-3.220**
2I47-B04	16	44	62	19	**-3.213**
3LEA-A	17	185	195	11	**-3.210**
6BE6-D04	19	261	276	16	**-3.205**
3LEA-A	20	185	195	11	**-3.189**

Table S2: Evaluation of quality of ADAM17 homology models. Analysis was performed by PROCHECK [2].

Serial number	Name of the model	Template name	Number of amino acids	Tool used for modelling	Stereochemical quality	
					Amino acids in most favoured region from Ramachandran plot	Over all G factor
**01**	ADAM17-extracellular domain model	Hybrid model **(Table S1)**	469 (215 - 684)	YASARA Structure version 17.8.19 [1]	85.3%	0.18
**02**	ADAM17-catalytic domain model	3LEA-A	260 (215-474)	YASARA Structure version 17.8.19 [1]	93.0%	0.21

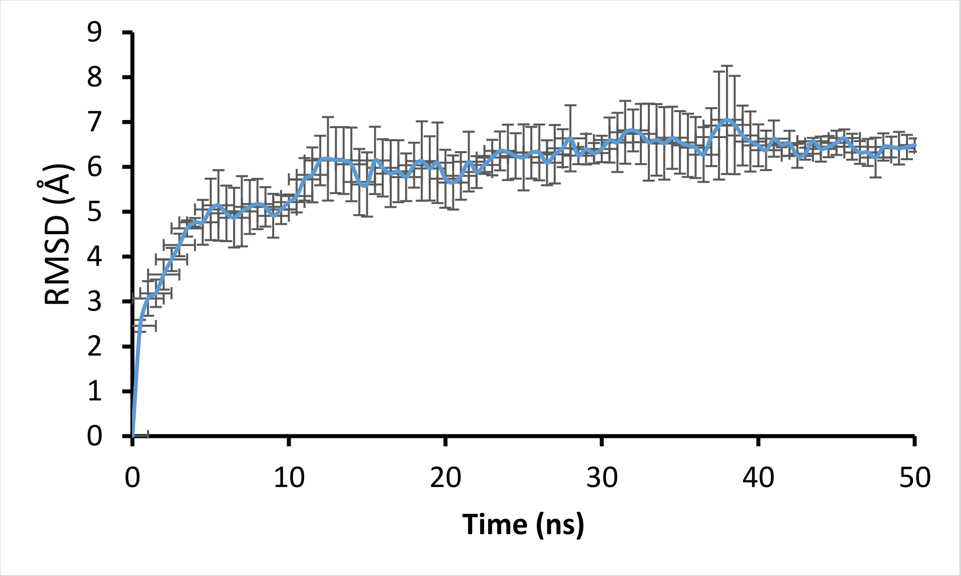

Figure S1: RMSD of extracellular domain model for 50 ns. MD Simulation by YASARA [3-5] was performed with model of the extracellular domain of ADAM17, constructed by YASARA. The time-average was build out of three independent MD runs each 50 ns long.

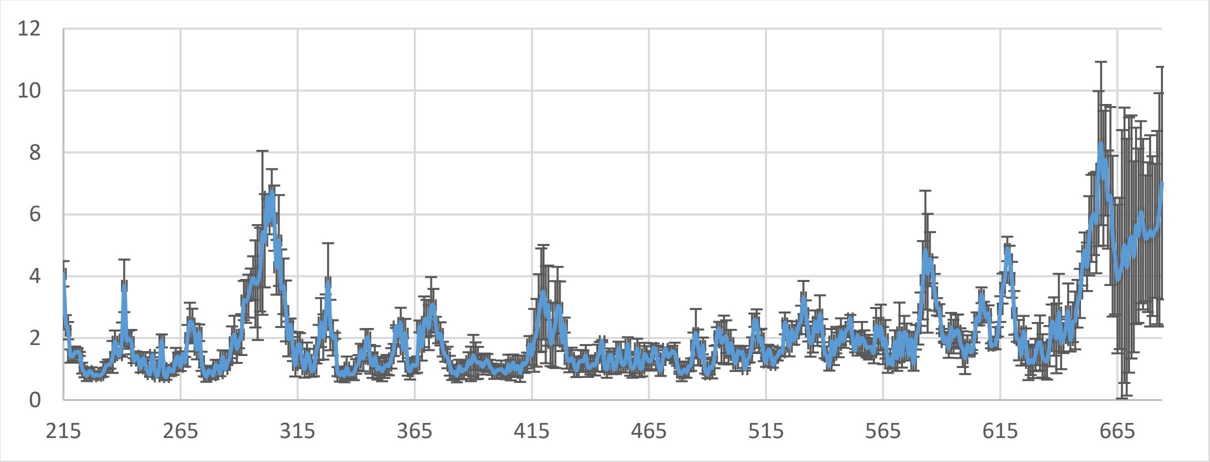

Figure S2: RMSF of extracellular domain model for 50 ns. MD Simulation by YASARA [3-5] was performed with model of the extracellular domain of ADAM17, constructed by YASARA. The time-average was build out of three independent MD runs each 50 ns long.

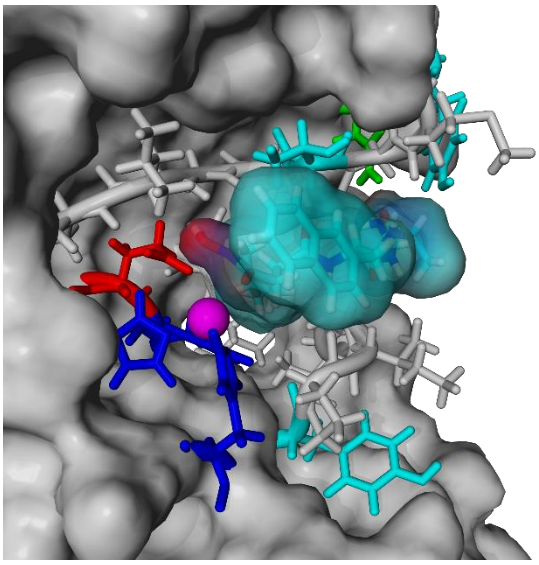

Figure S3: Molecular docking of TAPI-1 with the catalytic domain of ADAM17. The Inhibitor TAPI-1 (light blue molecular surface) was docked to the catalytic domain (grey molecular surface) and bound directly next to the bound zinc ion (magenta). First cluster of TAPI-1 with highest binding affinity was chosen. The coloring of the inhibitor surface indicates the atoms oxygen (red), nitrogen (blue) and carbon (cyan). All contact residues are colored as follows: Positive and negative charged amino acids are shown in blue and red, respectively. Residues in cyan mark AAs with hydroxyl groups, which can form hydrogen bonds. AAs in green mark polar uncharged amino acids. Hydrophobic and special AAs like Cys, Gly, and Pro are shown in grey.

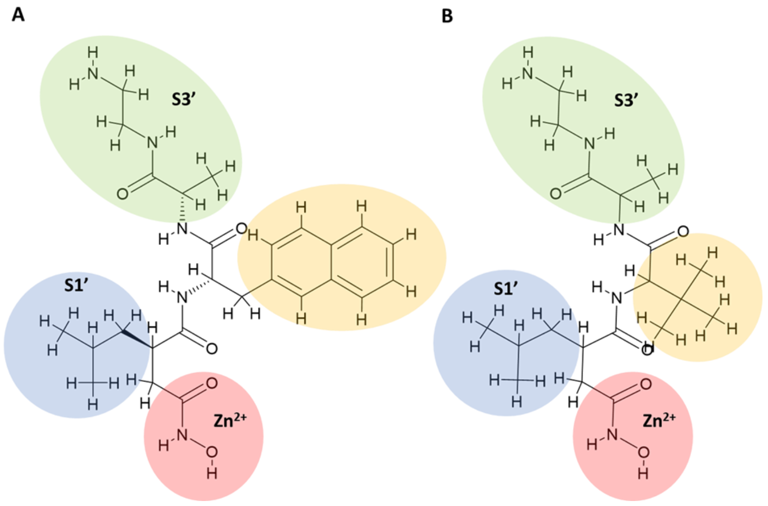

Figure S4: Hydroxamate-based inhibitors TAPI-1 and TAPI-2. Both inhibitors differ from each other only in one side group (yellow). TAPI-1 (**A**) and TAPI-2 (**B**) have one hydroxamate group (red) binding to zinc ions, one isobutyl group (blue), which binds the S1' pocket and one group binding to the S3' binding pocket in TACE proteases (green). TAPI-1: (2R)-N-[(2S)-1-[[(2S)-1-(2-aminoethylamino)-1-oxopropan-2-yl]amino]-3-naphthalen-2-yl-1-oxopropan-2-yl]-N'-hydroxy-2-(2-methylpropyl)butanediamide, TAPI-2: N-[1-[[1-(2-aminoethylamino)-1-oxopropan-2-yl]amino]-3,3-dimethyl-1-oxobutan-2-yl]-N'-hydroxy-2-(2-methylpropyl)butanediamide

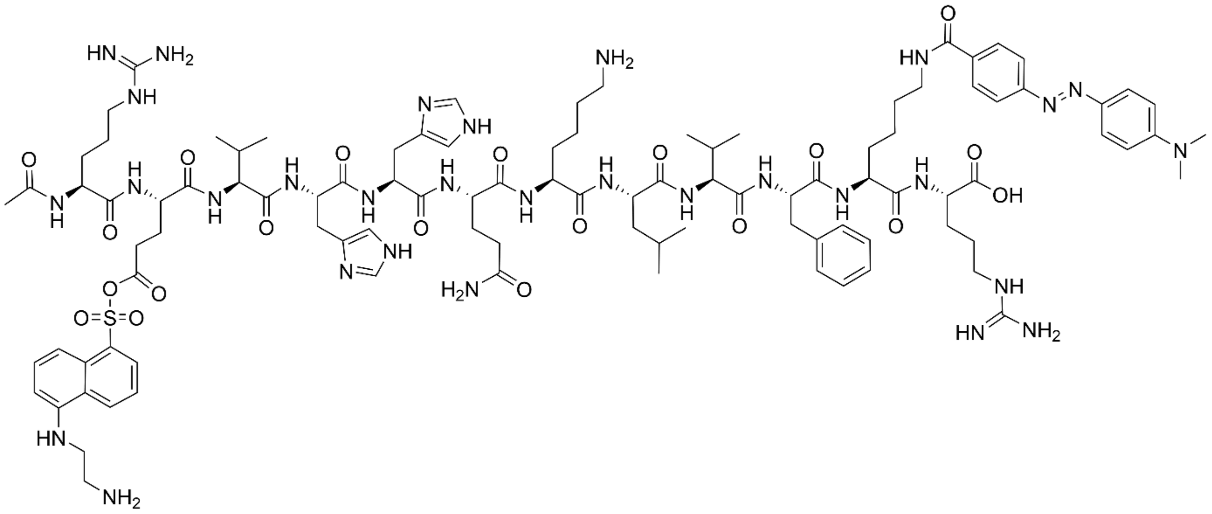

Figure S5: Structure of secretase substrate II (Ac-RE(EDANS)-VHHQKLVF-K(DABCYL)-R-OH). Ligand molecule was selected based on our previous reports [6].

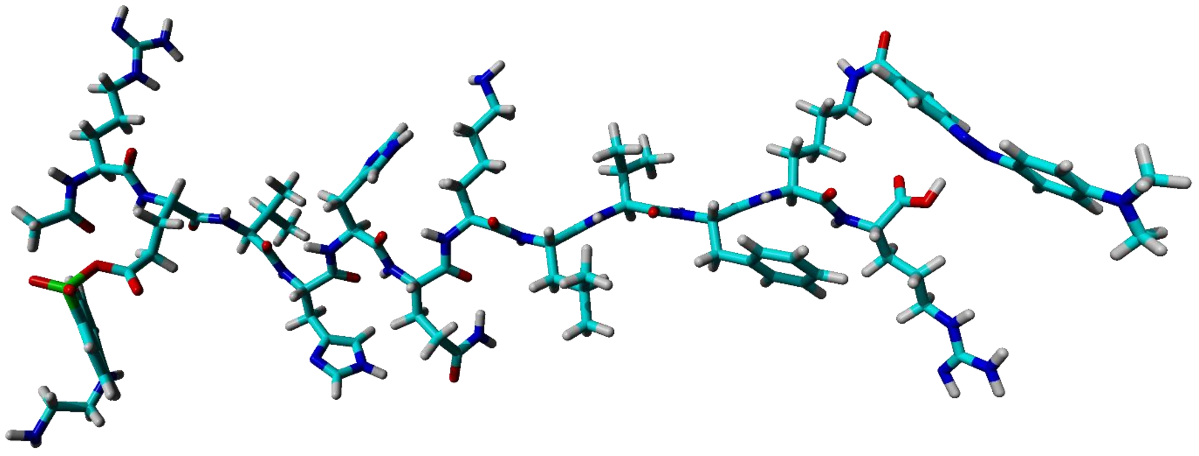

Figure S6: 3D structure of secretase substrate II Ac-RE(EDANS)-VHHQKLVF-K(DABCYL)-R-OH. Substrate for tumor necrosis factor-α converting enzyme (TACE) based on our previous reports [6].

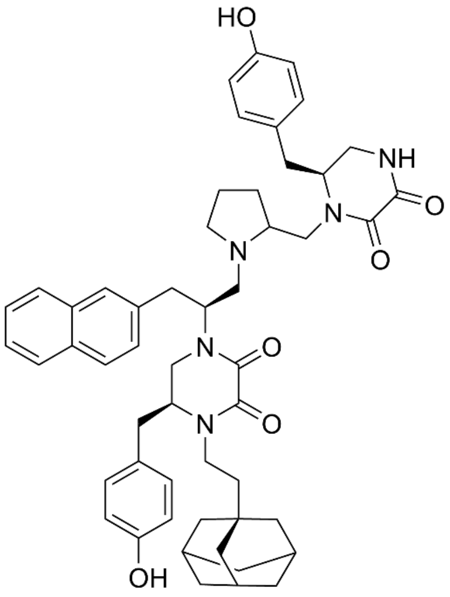

Figure S7: Structure of inhibitor molecule (CID17). (5S)-4-(2-(adamantan-1-yl)ethyl)-5-(4-hydroxybenzyl)-1-((2S)-1-(2-(((S)-6-(4-hydroxybenzyl)-2,3-dioxopiperazin-1-yl)methyl)pyrrolidin-1-yl)-3-(naphthalen-2-yl)propan-2-yl)piperazine-2,3-dione

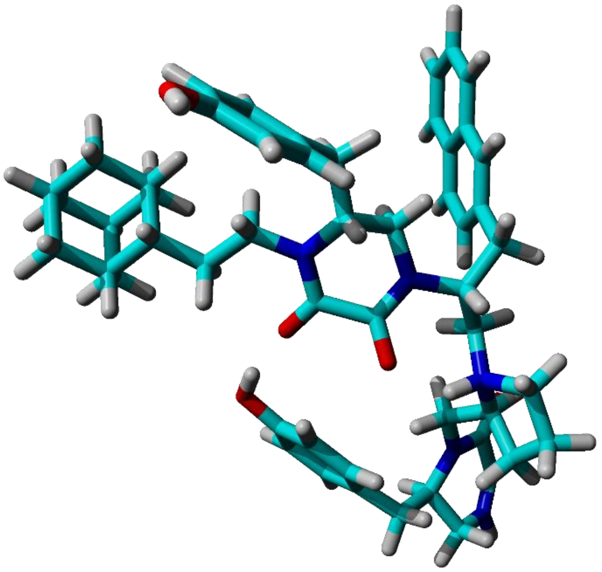

Figure S8: 3D structure of inhibitor molecule (CID17). Inhibitor used for molecular docking with ADAM17 protein model (215-684 AAs).

Table S3: Docking contact residues for different receptors and the used ligands. Contact residues were taken from the docking report. Underlined AAs are part of the extracellular domain linker, which is not the catalytic domain (475-684).

**Docking Receptor**	**Docking Ligand**	**Contact residues**
ADAM17 catalytic domain (R215-S474)	TAPI-1	MET 345 GLY 346 THR 347 LEU 348 GLY 349 LEU 350 ALA 351 ASN 389 TYR 390 GLU 398 LEU 401 VAL 402 HIS 405 GLU 406 HIS 409 HIS 415 TYR 436 PRO 437 ILE 438 ALA 439 VAL 440 ZN 476
ADAM17 catalytic domain (R215-S474)	Secretase II substrate	Glu 295 Lys 296 His 297 Lys 315 Leu 318 Glu 319 Gln 320 Ser 322 Phe 323 Asp 324 Met 345 Gly 346 Thr 347 Leu 348 Gly 349 Leu 350 Ala 351 Tyr 352 Val 353 Arg 357 Ser 360 His 361 Gly 362 Ser 371 Val 373 Ile 378 Leu 380 Tyr 390 Val 402 His 405 Glu 406 His 409 Ala 413 Glu 414 His 415 Asp 416 Pro 417 Asp 418 Tyr 436 Pro 437 Ile 438 Ala 439 Val 440 Zn 476
ADAM17 extracellular domain (R215-W684)	Exosite inhibitor (CID17)	Tyr 238 Met 239 Thr 246 Asn 249 Tyr 250 Ile 252 Glu 253 Asp 256 Arg 257 Thr 393 Ile 394 Leu 395 Thr 396 Lys 397 Ser 519 Pro 520 Cys 534 Gln 535 Glu 536 Ala 537 Ile 538 Thr 541 Cys 542 Lys 543 Cys 555 Pro 558 Leu 568 Gly 576 Lys 577 Cys 578
ADAM17 extracellular domain (R215-W684)	Secretase II substrate	Asp 313 Val 314 Lys 315 Thr 347 Gly 349 Leu 350 Ala 351 Tyr 352 Val 353 Asn 359 Ser 360 His 361 Gly 362 Ser 371 Pro 372 Leu 380 Glu 406 His 409 Glu 414 His 415 Asp 647 Ile 649 Phe 652 Trp 653 Ile 656 Asp 657 Asn 662 Thr 663 Phe 664 Gly 665 Lys 666 Phe 667 Leu 668 Ala 669 Asp 670 Asn 671 Ile 672 Val 673 Gly 674 Zn 802

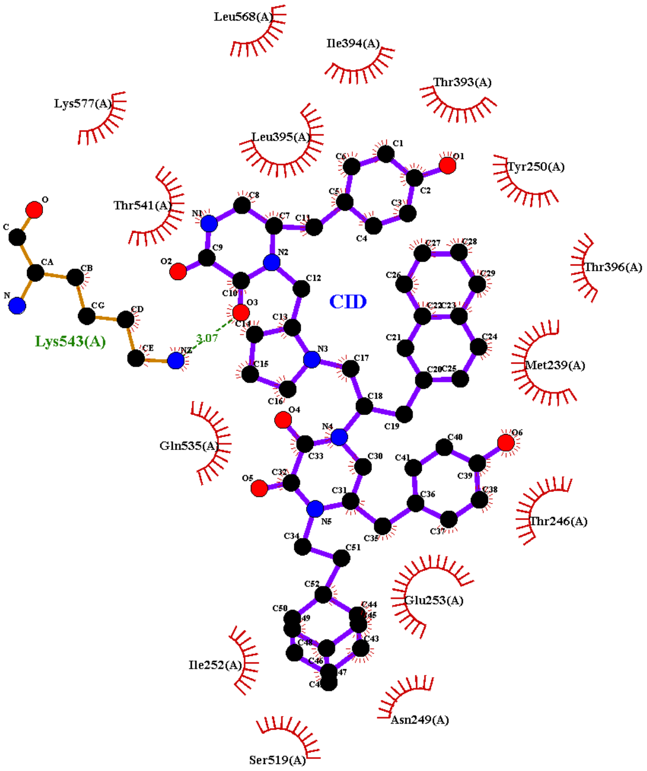

Figure S9: Visualization of key interactions towards bound inhibitor in exosite. Exosite inhibitor is mainly bound through hydrophobic interactions and one hydrogen bond between K543 and CID. Graphic visualization was done with LigPlot+ [7].

Text S1: Full-length sequence of human ADAM metallopeptidase domain 17 protein (gene ID: 6868, NP_003174.3, ADAM17). Prodomain are marked as underlined. Extracellular domain is the potential drug target and was used for 3D protein structure modelling as shown below.

>NP_003174.3 disintegrin and metalloproteinase domain-containing protein 17 preproprotein [*Homo sapiens*]

<u>MRQSLLFLTSVVPFVLAPRPPDDPGFGPHQRLEKLDSLLSDYDILSLSNIQQHSVRKRD</u>
<u>LQTSTHVETLLTFSALKRHFKLYLTSSTERFSQNFKVVVVDGKNESEYTVKWQDFFT</u>
<u>GHVVGEPDSRVLAHIRDDDVIIRINTDGAEYNIEPLWRFVNDTKDKRMLVYKSEDIK</u>
<u>NVSRLQSPKVCGYLKVDNEELLPKGLVDREPPEELVHRVKR</u>**RADPDPMKNTCKLL**
VVADHRFYRYMGRGEESTTTNYLIELIDRVDDIYRNTSWDNAGFKGYGIQIEQIR
ILKSPQEVKPGEKHYNMAKSYPNEEKDAWDVKMLLEQFSFDIAEEASKVCLAH
LFTYQDFDMGTLGLAYVGSPRANSHGGVCPKAYYSPVGKKNIYLNSGLTSTKNY
GKTILTKEADLVTTHELGHNFGAEHDPDGLAECAPNEDQGGKYVMYPIAVSGD
HENNKMFSNCSKQSIYKTIESKAQECFQERSNKVCGNSRVDEGEECDPGIMYLN
NDTCCNSDCTLKEGVQCSDRNSPCCKNCQFETAQKKCQEAINATCKGVSYCTG
NSSECPPPGNAEDDTVCLDLGKCKDGKCIPFCEREQQLESCACNETDNSCKVCC
RDLSGRCVPYVDAEQKNLFLRKGKPCTVGFCDMNGKCEKRVQDVIERFWDFID
QLSINTFGKFLADNIVGSVLVFSLIFWIPFSILVHCVDKKLDKQYESLSLFHPSNVEM
LSSMDSASVRIIKPFPAPQTPGRLQPAPVIPSAPAAPKLDHQRMDTIQEDPSTDSHMDE
DGFEKDPFPNSSTAAKSFEDLTDHPVTRSEKAASFKLQRQNRVDSKETEC

Text S2: Extracellular domain sequence of human ADAM metallopeptidase domain 17 protein (gene ID: 6868, NP_003174.3, ADAM17)*.

>NP_003174.3 disintegrin and metalloproteinase domain-containing protein 17 preproprotein [*Homo sapiens*]

(R)ADPDPMKNTCKLLVVADHRFYRYMGRGEESTTTNYLIELIDRVDDIYRNTSWDN
AGFKGYGIQIEQIRILKSPQEVKPGEKHYNMAKSYPNEEKDAWDVKMLLEQFSFDIA
EEASKVCLAHLFTYQDFDMGTLGLAYVGSPRANSHGGVCPKAYYSPVGKKNIYLNS
GLTSTKNYGKTILTKEADLVTTHELGHNFGAEHDPDGLAECAPNEDQGGKYVMYPI
AVSGDHENNKMFSNCSKQSIYKTIESKAQECFQERSNKVCGNSRVDEGEECDPGIMY
LNNDTCCNSDCTLKEGVQCSDRNSPCCKNCQFETAQKKCQEAINATCKGVSYCTGN
SSECPPPGNAEDDTVCLDLGKCKDGKCIPFCEREQQLESCACNETDNSCKVCCRDLS
GRCVPYVDAEQKNLFLRKGKPCTVGFCDMNGKCEKRVQDVIERFWDFIDQLSINTFG
KFLADNIVGSVLVFSLIFW

*Extracellular domain sequence of ADAM17 was used for 3D structure modeling. First amino acid was not included in the model.

Text S3: Catalytic domain sequence of human ADAM metallopeptidase domain 17 protein (gene ID: 6868, NP_003174.3, ADAM17). The catalytic domain is the potential drug target and was used for 3D protein structure modelling as shown below.

>NP_003174.3 disintegrin and metalloproteinase domain-containing protein 17 preproprotein [*Homo sapiens*]

RADPDPMKNTCKLLVVADHRFYRYMGRGEESTTTNYLIELIDRVDDIYRNTSWDNA
GFKGYGIQIEQIRILKSPQEVKPGEKHYNMAKSYPNEEKDAWDVKMLLEQFSFDIAEE
ASKVCLAHLFTYQDFDMGTLGLAYVGSPRANSHGGVCPKAYYSPVGKKNIYLNSGL
TSTKNYGKTILTKEADLVTTHELGHNFGAEHDPDGLAECAPNEDQGGKYVMYPIAV
SGDHENNKMFSNCSKQSIYKTIESKAQECFQERS

References

1. Krieger, E.; Joo, K.; Lee, J.; Lee, J.; Raman, S.; Thompson, J.; Tyka, M.; Baker, D.; Karplus, K. Improving physical realism, stereochemistry, and side-chain accuracy in homology modeling: Four approaches that performed well in CASP8. *Proteins* **2009**, *77 Suppl 9*, 114-122, doi:10.1002/prot.22570.
2. Laskowski, R.A.; Macarthur, M.W.; Moss, D.S.; Thornton, J.M. Procheck - a Program to Check the Stereochemical Quality of Protein Structures. *Journal of Applied Crystallography* **1993**, *26*, 283-291, doi:Doi 10.1107/S0021889892009944.
3. Berendsen, H.J.C.; Postma, J.P.M.; Vangunsteren, W.F.; Dinola, A.; Haak, J.R. Molecular-Dynamics with Coupling to an External Bath. *Journal of Chemical Physics* **1984**, *81*, 3684-3690, doi:Doi 10.1063/1.448118.
4. Krieger, E.; Vriend, G. YASARA View - molecular graphics for all devices - from smartphones to workstations. *Bioinformatics* **2014**, *30*, 2981-2982, doi:10.1093/bioinformatics/btu426.
5. Land, H.; Humble, M.S. YASARA: A Tool to Obtain Structural Guidance in Biocatalytic Investigations. *Methods Mol Biol* **2018**, *1685*, 43-67, doi:10.1007/978-1-4939-7366-8_4.
6. Knapinska, A.M.; Dreymuller, D.; Ludwig, A.; Smith, L.; Golubkov, V.; Sohail, A.; Fridman, R.; Giulianotti, M.; LaVoi, T.M.; Houghten, R.A.; et al. SAR Studies of Exosite-Binding Substrate-Selective Inhibitors of A Disintegrin And Metalloprotease 17 (ADAM17) and Application as Selective in Vitro Probes. *J Med Chem* **2015**, *58*, 5808-5824, doi:10.1021/acs.jmedchem.5b00354.
7. Laskowski, R.A.; Swindells, M.B. LigPlot+: multiple ligand-protein interaction diagrams for drug discovery. *J Chem Inf Model* **2011**, *51*, 2778-2786, doi:10.1021/ci200227u.
